# Overexpression of Soybean Isoflavone Reductase (*GmIFR*) Enhances Resistance to *Phytophthora sojae* in Soybean

**DOI:** 10.3389/fpls.2015.01024

**Published:** 2015-11-23

**Authors:** Qun Cheng, Ninghui Li, Lidong Dong, Dayong Zhang, Sujie Fan, Liangyu Jiang, Xin Wang, Pengfei Xu, Shuzhen Zhang

**Affiliations:** ^1^Key Laboratory of Soybean Biology of Chinese Education Ministry, Soybean Research Institute, Northeast Agricultural UniversityHarbin, China; ^2^Jiamusi Branch Academy of Heilongjiang Academy of Agricultural SciencesJiamusi, China; ^3^Heilongjiang Academy of Land Reclamation SciencesHarbin, China

**Keywords:** *Glycine max*, isoflavone reductase, *Phytophthora sojae*, isoflavonoid, gene expression, antioxidant properties

## Abstract

Isoflavone reductase (IFR) is an enzyme involved in the biosynthetic pathway of isoflavonoid phytoalexin in plants. IFRs are unique to the plant kingdom and are considered to have crucial roles in plant response to various biotic and abiotic environmental stresses. Here, we report the characterization of a novel member of the soybean isoflavone reductase gene family *GmIFR*. Overexpression of *GmIFR* transgenic soybean exhibited enhanced resistance to *Phytophthora sojae*. Following stress treatments, *GmIFR* was significantly induced by *P. sojae*, ethephon (ET), abscisic acid (placeCityABA), salicylic acid (SA). It is located in the cytoplasm when transiently expressed in soybean protoplasts. The daidzein levels reduced greatly for the seeds of transgenic plants, while the relative content of glyceollins in transgenic plants was significantly higher than that of non-transgenic plants. Furthermore, we found that the relative expression levels of reactive oxygen species (ROS) of transgenic soybean plants were significantly lower than those of non-transgenic plants after incubation with *P. sojae*, suggesting an important role of *GmIFR* might function as an antioxidant to reduce ROS in soybean. The enzyme activity assay suggested that GmIFR has isoflavone reductase activity.

## Introduction

A major response of soybean to attack by fungal pathogens and oomycetes is production of isoflavonoid phytoalexin glyceollins (Partridge and Keen, 1977; Yoshikawa et al., 1978; Banks and Dewick, 1983; Ng et al., 2011; Kim et al., 2012). They are valuable secondary metabolities produced primarily in leguminous plants and are rarely found in other plant families (Wang et al., 2006; Kim et al., 2010b), and are synthesized by the isoflavonoid branch of the central phenylopropanoid pathway (Ng et al., 2011). Moreover, glyceollins in general protect plant tissues from environmental challenge possibly by reducing the oxidative damage induced by stress factors; therefore, the compounds can possess considerable cellular antioxidant properties (Nwachukwu et al., 2013). A huge variety of enzymes are thought to be involved in their biosynthetic pathways (Somerville and Somerville, 1999). Isoflavone reductase (IFR) is identified as a crucial enzyme involved in the synthesis of the glyceollins from daidzein (Graham et al., 1990; Oliver et al., 2003), and catalyzes a stereo-specific NADPH-dependent reduction to (3R)-isoflavanone (Guo et al., 1994; Cooper et al., 2002). In addition, IFR is a monomeric, cytosolic reductase, and the enzyme can use 2′-hydroxydaidzein, 2′-hydroxyformononetin, and 2′-hydroxygenistein as substrates in soybean (Wang et al., 2006).

IFRs are members of a large protein family, and their cDNAs cloned from leguminous plants such as alfalfa (*Medicago sativa*; Paiva et al., 1991), pea (*Pisum sativum*; Sun et al., 1991), kidney bean (*Phaseolus vulgaris*; Rípodas et al., 2013) share high levels of sequence identity, and they participate specifically in isoflavonid phytoalexin biosynthesis. IFR was first identified as a key enzyme involved in the latter part of the isoflavonoid phytoalexins pathway in alfalfa (Paiva et al., 1991). In pea, the enzyme has been purified and used to raise polyclonal antibodies (Sun et al., 1991). The levels of IFR in response to *Ascochyta rabiei* in chickpea were higher than those of control, suggesting that IFR may play a role in determining resistance to fungal (Daniel et al., 1990). In kidney bean, the reduction of IFR levels affected the growth, lateral root elongation and the number of nodules developed (Rípodas et al., 2013). However, beyond the purification to apparent homogeneity of IFR from elicitor-challenged soybean cell cultures and some physical and kinetic properties of this enzyme (Fischer et al., 1990; Wang et al., 2006), the gene function is still unclear in soybean.

In addition, IFR-like (IRL) proteins from non-leguminous plants, which are also part of the IFR family (Kim et al., 2010b), have also been cloned from tobacco (*Nicotiana tabacum*; Shoji et al., 2002), rice (*Oryza sativa*; Kim et al., 2003), Arabidopsis (Babiychuk et al., 1995), Ginkgo (*Ginkgo biloba*; Cheng et al., 2013), etc. They show amino acid sequence homology with those of legumes IFRs. Moreover, several IRL proteins have been implicated in response to biotic or abiotic stresses (Babiychuk et al., 1995; Lers et al., 1998; Shoji et al., 2002; Kim et al., 2003, 2010b). For instance, *OsIRL* gene was induced with an expression pattern similar to methyl jasmonic acid (MeJA) induction after *Pyricularia grisea* inoculation of rice (Kim et al., 2003). It has been proposed that AtIRL might have an important role as an antioxidant in yeast (Babiychuk et al., 1995). A recent study showed that OsIRL may behave as an antioxidant in response to reactive oxygen species (ROS) in suspension-cultured cells as well as during root development in rice (Kim et al., 2010b).

In a previous study, a cDNA library enriched for mRNAs encoding ESTs that increased in abundance during infection with *Phytophthora sojae* was constructed by suppression subtractive hybridization from leaf tissues of a high resistant soybean cultivar “Suinong 10,” and an EST homologous to an isoflavone reductase from white lupin (*Lupinus albus*) was identified to be up-regulated by microarray and real-time PCR (Xu et al., 2012). In this study, the full-length EST, designated *GmIFR* (GenBank accession no. NM_001254100, NCBI protein no. NP_001241029), was isolated through RT-PCR from “Suinong 10” soybean, and the transgenic soybean plants over-expressing *GmIFR* gene under the control of 35S promoter were produced. The expression patterns of *GmIFR* induced under biotic stresses were also examined. Moreover, the function of *GmIFR* and the content of daidzein, genistein, glycitein, the relative content of glyceollins and the ROS in transgenic plants were investigated. GmIFR protein could catalyze a distinct NADPH-dependent oxidoreductase reaction by enzyme activity assay, suggesting that GmIFR has isoflavone reductase activity. Together, we report insights into the function of an isoflavone reductase (IFR) in soybean, namely GmIFR, in host responses to *P. sojae*.

## Materials and methods

### Plant materials and stress treatments

The soybean cultivar “Suinong10,” resistant to the predominant race 1 of *P. sojae* in Heilongjiang, China (Zhang et al., 2010), was used in this study. The seeds of “Suinong10” were grown with a photoperiod of 16/8 h light/dark and maintained at 22°C with 70% relative humidity in the greenhouse. Fourteen days after planting, seedlings at the first-node stage (V1; Fehr et al., 1971) were used for various treatments.

For abiotic treatments, the seedlings were exposed to one of the five different stresses, namely wounding, ET, ABA, SA, or MeJA. For wounding treatment, the edges of leaves were cut by about 0.2 cm with scissors and incubated at room temperature for 0, 1, 3, 6, 9, 12 or 24 h; the intact leaves of soybean were used as controls. The ABA (200 μM), SA (2 mM), and MeJA (100 μM) were dissolved in 0.01% Tween 20 and sprayed onto young leaves for 0, 1, 3, 6, 9, 12 or 24 h. Ethylene treatment was performed with a concentration of 200 μL L^−1^ by injection of gaseous ethylene in a sealed plexiglass chamber for 0, 1, 3, 6, 9, 12, or 24 h. The control leaves were sprayed with an equivalent volume of 0.01% (v/v) Tween 20.

For *P. sojae* treatment, the soybean plants were inoculated with *P. sojae* zoospores following the method described by Ward et al. (1979) and Morris et al. (1991) with minor modifications. Mock inoculations were carried out with equivalent amounts of sterile water. Zoospores were developed with the procedure of Ward et al. (1979), and the concentration was estimated using hemacytometer to approximately 1 × 10^5^ spores mL^−1^. The unifoliolate leaves were also treated for 0, 3, 6, 9, 12, 24, 36, 48, 64, 72 h. “Dongnong50” soybean, which was susceptible to *P. sojae* race 1, obtained from the Key Laboratory of Soybean Biology in Chinese Ministry of Education, Harbin, was used for gene transformation experiments.

### Isolation of the GmIFR gene

A suppression subtractive hybridization library coupled with cDNA microarrays was queried using a soybean EST encoding an EST homologous to an isoflavone reductase from white lupin (*L. albus*), previously shown to be up-regulated in “Suinong 10” soybean inoculated with *P. sojae* (Xu et al., 2012). Here, the full-length cDNA (tremed *GmIFR*, GenBank accession no. NM_001254100, NCBI protein no. NP_001241029) of the EST was amplified by RT-PCR from cDNA of “Suinong 10” using the primer pairs *GmIFRF* and *GmIFRR* (Supplementary Table S1). PCR was performed as follows: 94°C for 5 min, followed 30 cycles of 94°C for 30 s, 60°C for 30 s, and 72°C for 90 s, with a final extension at 72°C for 10 min. The amplification product was gel purified and cloned into the pMD18-T vector (TaKaRa, Dalian, China). An analysis of protein structure was performed using Smart (http://smart.embl-heidelberg.de/). Sequence alignments were performed using DNAMAN software (http://www.lynnon.com/). A phylogenetic analysis of *GmIFR* and various heterologous IFR proteins was performed using MEGA4 software (Tamura et al., 2007).

### Quantitative RT-PCR analysis

Quantitative real-time PCR analysis was performed to determine the transcript abundance of *GmIFR*. Total RNA was isolated from “Suinong 10” soybean leaves using Trlzol reagent (Invitrogen, Shanghai, China). The synthesis of cDNA was conducted using an oligo(dT) primer and a M-MLV reverse transcriptase kit (Takara, Dalian, China) according to the manufacturer's instructions. qRT-PCR was performed on a CFX96 Touch™ Real-Time PCR machine (Biobad, USA) using the real-time PCR kit (ToYoBo, Japan). DNA accumulation was measured using SYBR Green as the reference dye. The soybean housekeeping gene *Gmactin4* (GenBank accession no. AF049106) was used as the internal control (see Supplementary Table S1 for primer sequences). For tissue distribution analysis, the transcript level of *GmEF1* gene (GenBank accession no. NM_001248778) was used as quantitative control (see Supplementary Table S1 for primer sequence). The relative expression of target gene in different tissues of soybean was calculated using the 2^−ΔΔCT^ method. For each sample, three biological replicates were analyzed with their respective technical replicates.

### Subcellular localization of GmIFR protein

To determine the subcellular localization of GmIFR, the coding region of GmIFR was fused to the N-terminus of GFP under the control of the CaMV 35S promoter in the PCAMBIA1302 vector. Soybean protoplasts were obtained according to the method described by Lin (1983). Soybean protoplast transformation described by Yoo et al. (2007) was performed with minor modifications.

### Expression and purification of fusion protein

The full-length cDNA of *GmIFR* was fused to the N-terminus of the 6 × His-tag, at the *Nco*I and XhoI restriction sites of the vector pET28a (+) (Novagen, Germany). The recombinant fusion plasmid was expressed into *Transetta* (DE3) cells (TransGen Biotech, China). His-tagged proteins were induced with 0.5 mM isopropyl-β-D-thiogalactoside (IPTG) at 37°C for 4 h. The fusion protein was purified at room temperature and quantified according to the pET System Manual (Novagen). The fusion GmIFR protein was subsequently analyzed by sodium dodecyl sulfate polyacrylamide gel electrophoresis (SDS-PAGE) and western blotting using anti-His antibody.

### Enzyme assays

In order to determine whether GmIFR has isoflavone reductase activity, the enzyme assays of GmIFR was analyzed. The fusion protein was used in the enzyme assays. GmIFR activity was analyzed according to the method described by Paiva et al. (1991) with minor modifications. High Performance Liquid Chromatography (HPLC) was used to separate the substrate and product.

### Plasmid construction and transformation of soybean

For gene overexpression analysis, the full length *GmIFR* coding region was amplified with gene specific primers *GmIFRTF* and *GmIFRTR* (Supplementary Table S1). The PCR conditions were as follows: 94°C for 2 min followed by 30 cycles at 94°C for 30 s, 55°C for 30 s, and 72°C for 1 min and a final extension at 72°C for 10 min. Then the *GmIFR* open reading frame was cloned into the vector pCAMBIA3301 under the control of a CaMV35S promoter. The constructs were transferred into the *Agrobacterium tumefaciens* strain LBA4404 via tri-parental mating. For “Dongnong 50” soybean transformation, the cotyledonary nodes were used as explants for the transformation using the *Agrobacterium*-mediated transformation method described by Paz et al. (2004). Transgenic soybean plants (T7) were indentified by PCR amplification and southern hybridization using the DIG High Prime DNA Labeling and Detection Starter Kit II (Roche Cat., Germany), and they were developed to T8 transgenic soybean plants for further analysis.

### Pathogen response assays of transgenic soybean plants

To investigate whether the *GmIFR*-transformed plants resist pathogen infection, artificial inoculation procedures were performed according to the methods described by Dou et al. (2003) and Morrison and Thorne (1978) with some modifications. The living cotyledons of three T8 transgenic soybean plants (numbered T8-80, T8-88, and T8-96) were treated with a *P. sojae* inoculum. For infection assays, three biological replicates were analyzed with their respective technical replicates. The living cotyledons were incubated in a mist chamber at 25°C with 90% relative humidity under a 14 h photoperiod at a light intensity of 350 μmol photons m^−1^ s^−1^ for investigation. The cotyledons of non-transformed plants were used as controls. Disease symptoms on each cotyledon were observed and photographed after inoculation using a Nikon D7000 camera.

### Measurement of reactive oxygen species (ROS) generation

To investigate whether the *GmIFR*-transformed plants respond to oxidative stresses, the hypocotyls of three T8 transgenic soybean plants (numbered T8-31, T8-39, and T8-47) and non-transgenic soybean plants were treated with *P. sojae* zoospores of approximately 1 × 10^5^ spores mL^−1^ for 0, 3, 6, 12, 24, 48 h following the method described by Ward et al. (1979) and Morris et al. (1991) with minor modifications. The ROS were measured according to the instructions supplied with the Reactive Oxygen Species Assay Kit (Beyotime Institute of Biotechnology, Haimen, China). In this kit, the non-fluorescent probe 2′,7′-dichlorofluorescein diacetate (H2DCF-DA) passively diffuses into cells and is deacetylated to form nonfluorescent 2′,7′-dichlorofluorescein (DCFH). DCFH reacts with ROS to form the fluorescent product DCF, which is trapped inside the cells. Fluorescence was detected at 485 nm for excitation and 530 nm for emission with a fluorescence microplate reader (Bio-TEK, USA; Qian et al., 2009).

### Isoflavone and glyceollins analysis

Approximately 0.1 g sample of seeds (numbered S-T8-80, S-T8-88, S-T8-96) developed from T8-80, T8-88 and T8-96 transgenic soybean plants, and that of non-transgenic soybean plants were ground to a fine power using a commercial coffee grinder. Daidzein, genistein, and glycitein were extracted from flour and separated using HPLC as described previously (Zeng et al., 2009). Measurements were done as micrograms of isoflavone per gram of seeds plus and minus the standard deviations.

Glyceollins were extracted from the seeds (namely S-T8-80, S-T8-88, S-T8-96) developed from T8-80, T8-88, and T8-96 transgenic soybean plants and non-transgenic soybean with 80% ethanol following the method described by Boue et al. (2000) and isolated using HPLC as described previously (Zeng et al., 2009).

## Results

### Isolation and bioinformatic analysis of GmIFR

The full-length cDNA sequence of *GmIFR* (GenBank Accession No. NM_001254100) was isolated from total RNA of “Suinong10” soybean by RT-PCR and cloned into pMD-18T vector. Sequence analysis showed that *GmIFR* has an open reading frame (ORF) of 939 bp and encodes a polypeptide of 312 amino acids (Supplementary Figure S1) with a predicted molecular mass of 34.92 kDa and a isoelectric point (pI) of 6.33. The deduced protein has a central 107 amino acid NAD (P) domain (Supplementary Figure S1). The predicted three-dimensional model of the GmIFR consists of 13 α-helices and 10 β-strands (Supplementary Figure S2A). To further explore the evolutionary relationship among plant NAD (P) proteins, a phylogenetic tree was constructed using MEGA 4.0 (Tamura et al., 2007) based on the amino acid sequences. Alignment and phylogenetic tree analysis of leguminous plants sequences revealed that GmIFR has 44–94% identity for overall amino acid sequence to *Phaseolus vulgaris* PvIFR, *Cicer arietinum* CaIFR, *Medicago truncatula* MtIRF, *Lotus japonicus* LjIFR, *Glycine soja* GsIFR, *Medicago sativa* MsIFR, *Pisum sativum* PsIFR (Supplementary Figure S2B). Analysis of the conserved NAD (P) domain of 107 aa showed that GmIFR shared 86–90% amino acid identity with others (Supplementary Figure S2C). The analysis of homologs of *GmIFR* in the soybean genome, based on data obtained from the Phytozome database (http://www.phytozome.net/ soybean), indicated that the two genes were clustered on two linkage groups, namely one each on Gm 01 and Gm 09 and had three introns.

### Accumulation of the GmIFR transcript under different stress treatments

**Figure 1:**
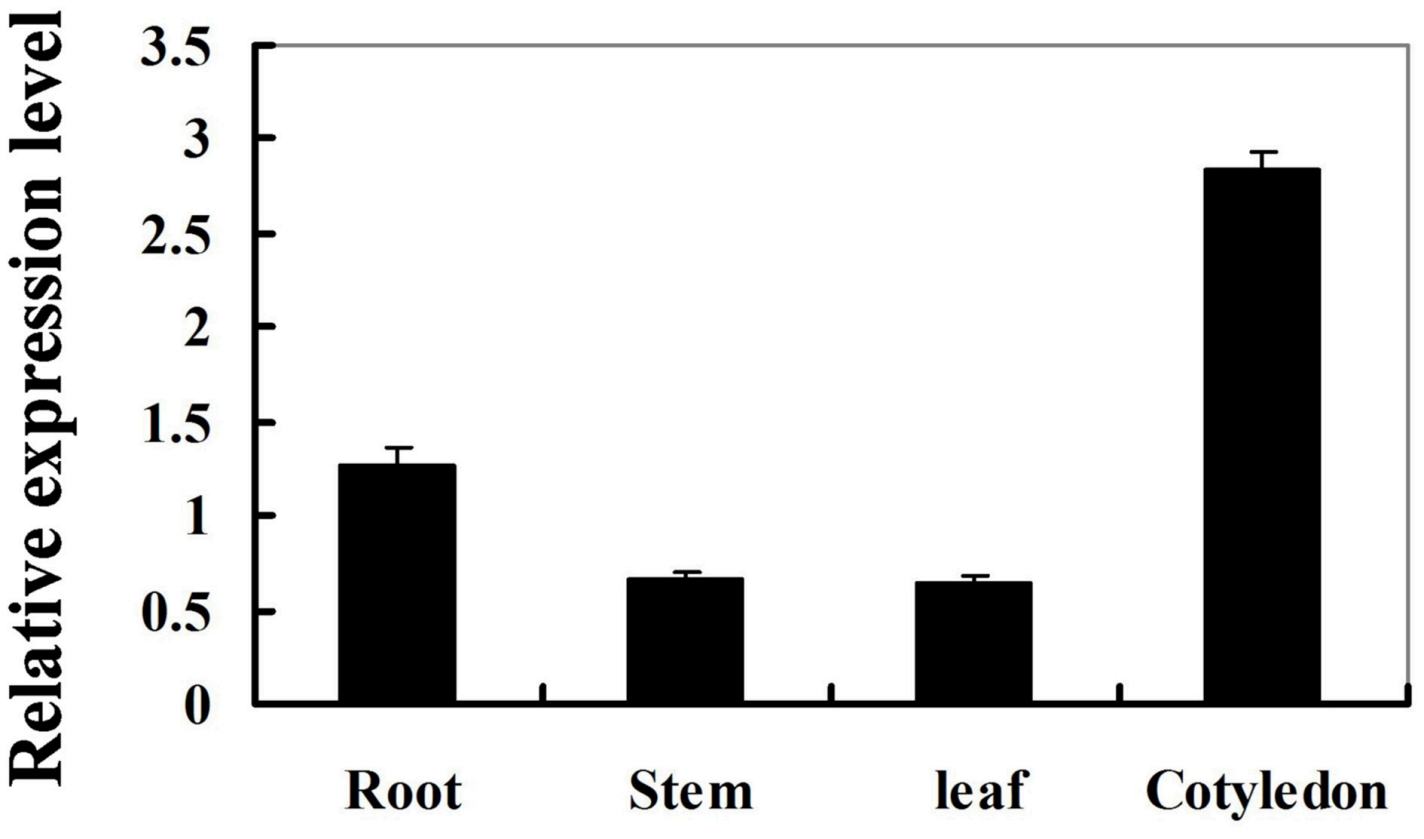
**Expression patterns of the *GmIFR* genes in various tissues of “Suinong” 10 soybean under normal condition**. The roots, stems or leaves were prepared from 14-day-old seedlings, and the cotyledons from 7-day-old seedlings. The amplification of the soybean *EF1* (*GmEF1*) gene was used as an internal control to normalize all the data. For each sample, three biological replicates were analyzed with their respective three technical replicates, bars indicate standard error of the mean.

**Figure 2:**
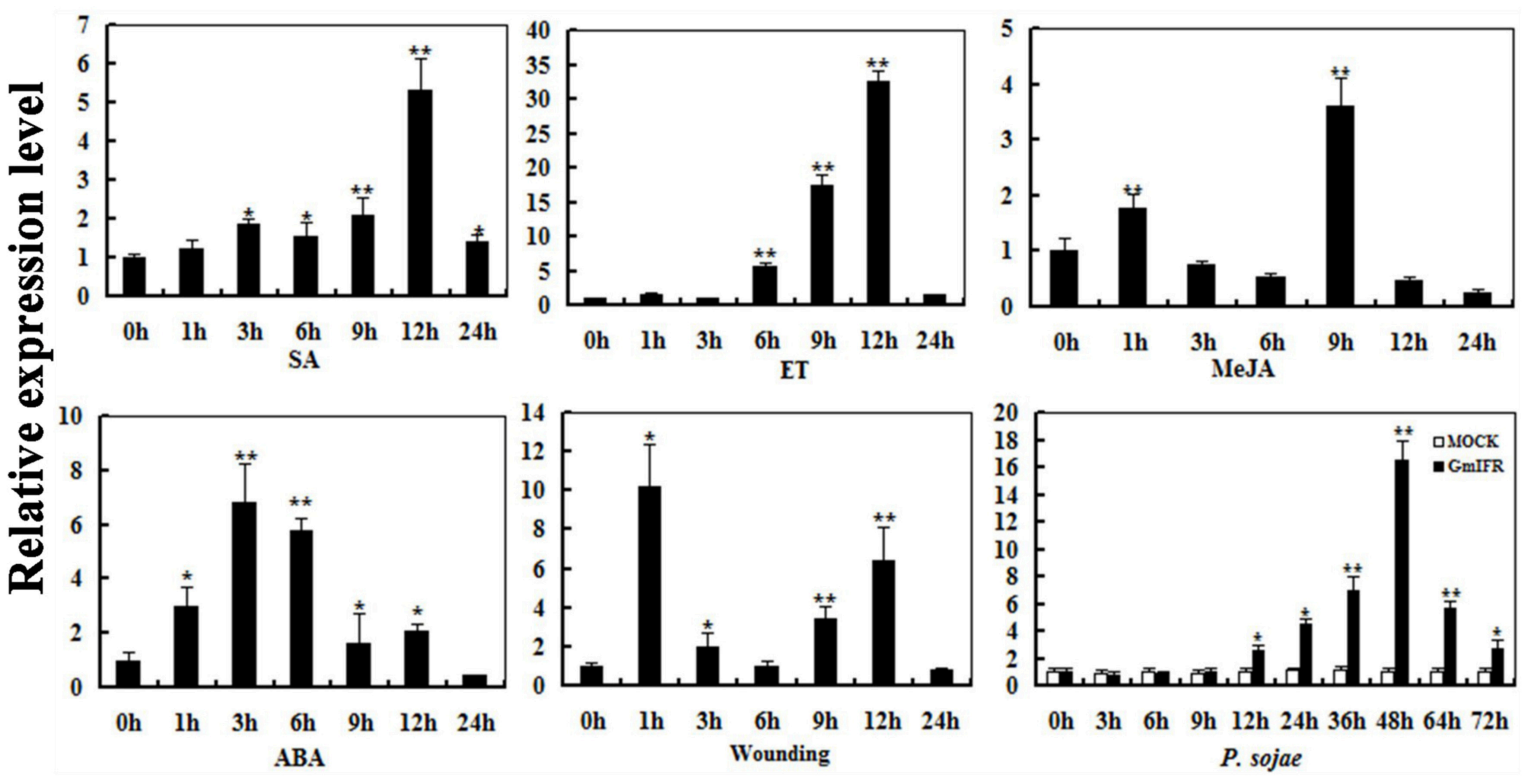
**Relative quantities of *GmIFR* mRNA at various time points post-treatment with ET, SA, MeJA, ABA, wounding, and *P. sojae***. Fourteen-day-old plants were used for treatments and analyses. Water treatment were used as control for *P. sojae* treatments. The amplification of the soybean *Actin* (*GmActin4*) gene was used as an internal control to normalize all the data. Relative transcript levels of *GmIFR* were quantified compared with mock plants at the same time point. The experiment was performed on three biological replicates with their respective three technical replicates and statistically analyzed using Student's *t*-test (^*^P < 0.05; ^**^P < 0.01). Bars indicate standard error of the mean.

To determine the expression pattern of *GmIFR*, quantitative real-time reverse transcription-PCR (qRT-PCR) was performed to examine the transcript levels of *GmIFR* in “Suinong 10” soybean plant. The examination of tissue-specific transcript abundance in “Suinong 10” soybean showed that *GmIFR* was constitutively and highly expressed in the cotyledons, followed by roots, stems, and leaves (Figure 1). Quantitative real-time PCR showed that *GmIFR* was responsive to ET, SA, MeJA, ABA, wounding and *P. sojae* treatments (Figure 2). *GmIFR* mRNA rapidly increased under ET and SA treatments, reaching a maximum level at 12 h after the treatment followed by a rapid decline (Figure 2). Under wounding, ABA, MeJA, and *P. sojae* treatments, *GmIFR* mRNA accumulated and reached a maximum level at 1, 3, 9 and 48 h, respectively (Figure 2).

### Subcellular localization of the GmIFR protein

**Figure 3:**
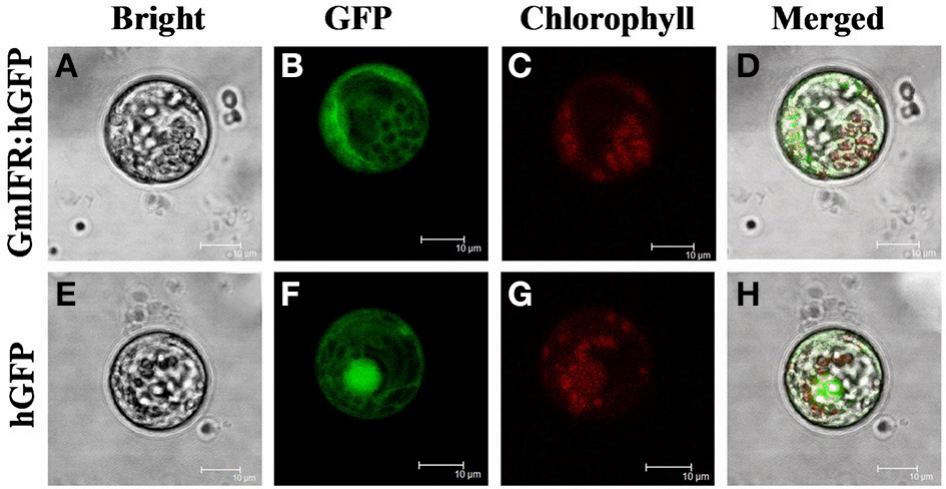
**Subcellular localization of the IFR-GFP fusion protein in Soybean protoplasts**. GmIFR-GFP expression was driven by the cauliflower mosaic virus 35S promoter and transiently expressed in Soybean protoplasts. The images of bright-field **(A,E)**, the GFP fluorescence (green) only **(B,F)**, the chlorophyll autofluorescence (red) only **(C,G)**, cytoplasmic marker fluorescence localization, and combined ones **(D,H)** are shown. Bars = 10 mm.

The Psort program predicted a cytoplosmic localization of GmIFR with 45% certainty (http://psort.hgc.jp/form.html). To determine the subcellular localization of GmIFR protein, GmIFR-GFP (for green fluorescent protein) fusion protein driven by the cauliflower mosaic virus 35S promoter was introduced into soybean protoplast by transient transformation. Analysis of the subcellular localization of the GmIFR-GFP fusion proteins by confocal laser scanning microscopy revealed that a strong fluorescent signal derived from GFP alone was observed in the cytoplasm, nuclei and cell membrane, whereas transformed cells carrying GmIFR-GFP showed a strong green fluorescence signal in the cytoplasm (Figure 3), demonstrating the cytoplasm localization of GmIFR.

### The activity of GmIFR *in vitro*

**Figure 4:**
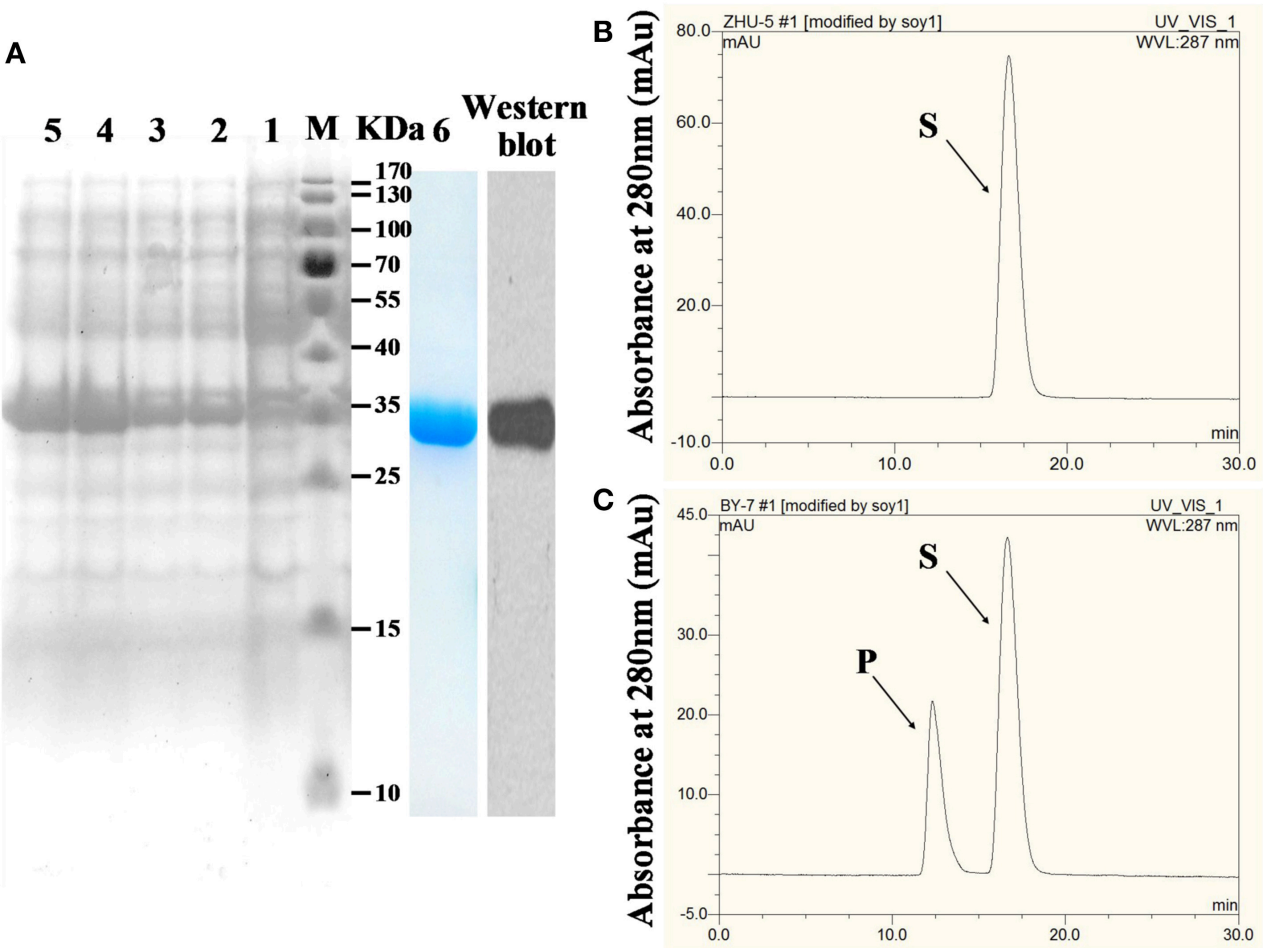
**Analysis of GmIFR activity by HPLC**. **(A)** After IPTG induction, *Transetta* cells containing pET28a-IFR were grown at 37°Cfor 1, 2, 4, 6 h. Lane *1* protein of total cells without IPTG induction, lane 2 protein of total cells with IPTG induction for 1 h, lane 3 protein of total cells with IPTG induction for 2 h, lane 4 induction for 4 h, lane 5 induction for 6 h, lane 6 purified recombinant GmIFR protein with Nickel-CL agarose affinity chromatography and used for enzyme activity assay; *M*, molecular marker; *Lane Western blot* western blotting of the purified recombinant GmIFR protein with an anti-His tag primary antibody probe. **(B)** The reaction with 2′-hydroxyformononetin. **(C)** The reaction harboring the GmIFR protein and with 2′-hydroxyformononetin as the substrate. S, substrate; P, product.

To express GmIFR in *Transetta* (DE3) cells, the coding sequence of *GmIFR* was cloned into pET-28a that was an expression vector with a His-tag. Upon induction by IPTG, GmIFR was expressed as a major soluble protein product at 1, 2, 4, 6 h (Figure 4A, Lane 2, 3, 4, 5). The molecular weight of the purified protein was about 34 kDa in SDS-PAGE, consistent with the calculated molecular mass (34 kDa) (Figure 4A, lane 6). Western blotting of the purified recombinant GmIFR protein confirmed its specific immune reactivity to anti-His antibodies (Figure 4A, lane Western blot).

In order to determine whether GmIFR has isoflavone reductase activity, the enzyme assays of GmIFR was analyzed. The reductase reactions in the presence of NADPH were measured by reversed-phase HPLC: the formation of the product from the substrate. As shown in Figures 4B,C, the protein purified from *Transetta* (DE3) expressing the fusion protein showed clear isoflavone reductase activity, with 2′-hydroxyformononetin as the substrate. These results proved that GmIFR is an isoflavone reductase.

### Overexpression of *GmIFR* enhanced resistance to *P. sojae* in soybean

**Figure 5:**
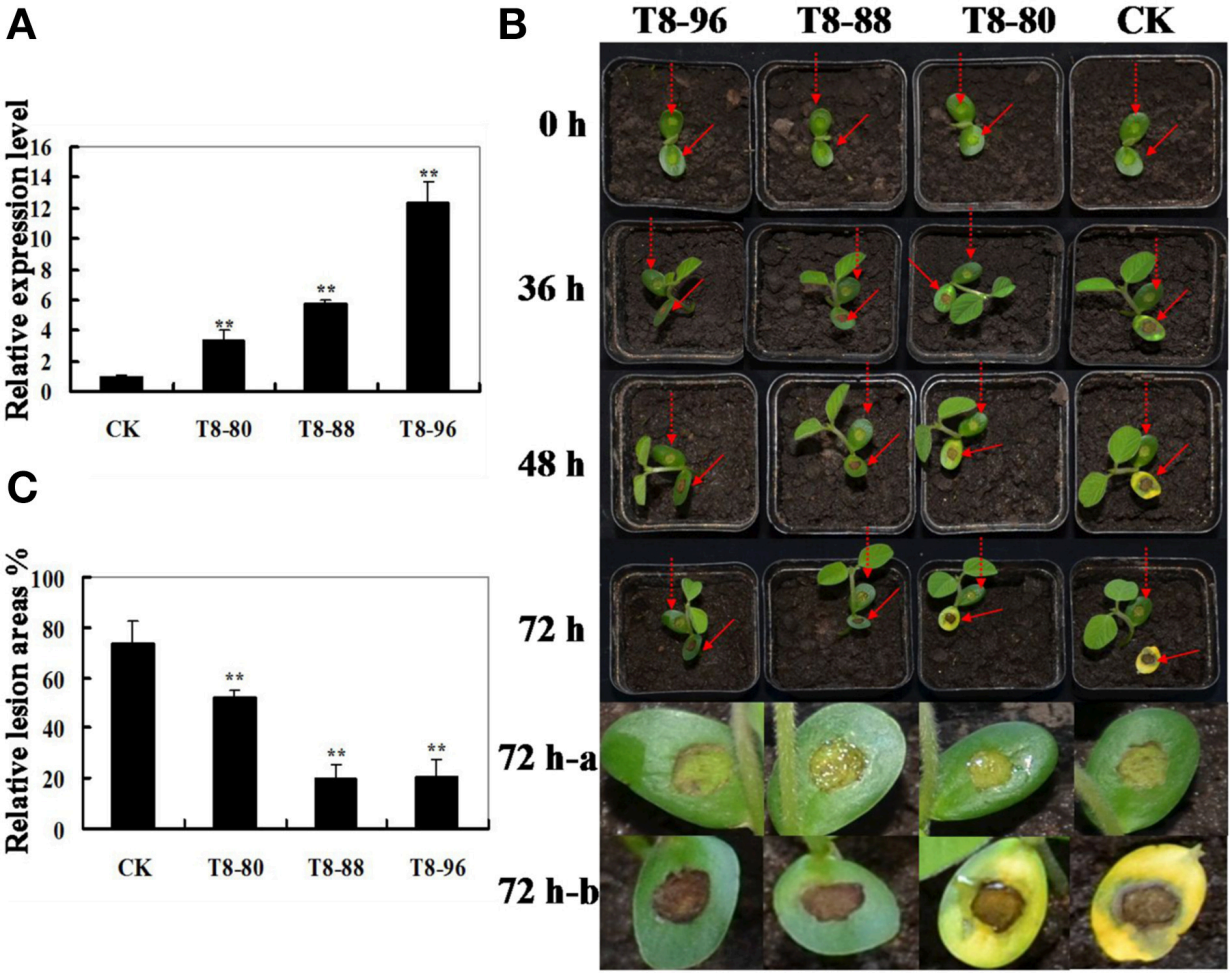
**Resistance analysis of *GmIFR* transgenic soybean plants**. **(A)** qRT-PCR determining the relative abundance of *GmIFR* (lines T8-80, T8-88, and T8-96) in the transgenic soybean plants. The non-transgenic soybean plants were used as controls. For each sample, three biological replicates were analyzed with their respective three technical replicates and statistically analyzed using Student's *t*-test (^**^P < 0.01). Bars indicate standard error of the mean. **(B)** Disease symptoms after infection with *P. sojae*. Lesions on living cotyledon at 72 h with *P. sojae* isolate race 1. For infection assays, three biological replicates were analyzed with their respective technical replicates. And do the same with control. **(C)** Relative lesion area of transgenic soybean cotyledon infection with *P. sojae* after 72 h. Seventy-two hours a represents the cotyledon of transgenic soybean and non-transgenic soybean infected with V8 juice agar, and 72 h-b represents the cotyledon were treated with a *P. sojae* inoculum. The average lesion area of each independent transgenic line (*n* = 3) was calculated and their relative lesion areas are shown in columns after comparison with the average lesion area on non-transgenic soybean. The statistically analyzed using Student's *t*-test (^**^P < 0.01). Bars indicate standard error of the mean.

To investigate whether overexpression of *GmIFR* in soybean has an effect on Phytophthora root rot resistance, the living cotyledons of three T8 transgenic soybean plants (T8-80, T8-88, T8-96) were selected by Real-time PCR (Figure 5A).

As shown in Figure 5B, the cotyledons of the non-transgenic soybean plants detached and exhibited clear and large water-soaked lesions compared with those of the transgenic plants after 72 h of incubation with *P. sojae* (Figure 5B). The lesion area of the three transgenic lines was significantly (*P* < 0.01) smaller than that of non-transgenic soybean plants at 72 h after inoculation (Figure 5C). These results indicated that constitutive expression of the *GmIFR* enhances resistance toward *P. sojae*.

### Expression of GmIFR in soybean seed affects ROS levels

**Figure 6:**
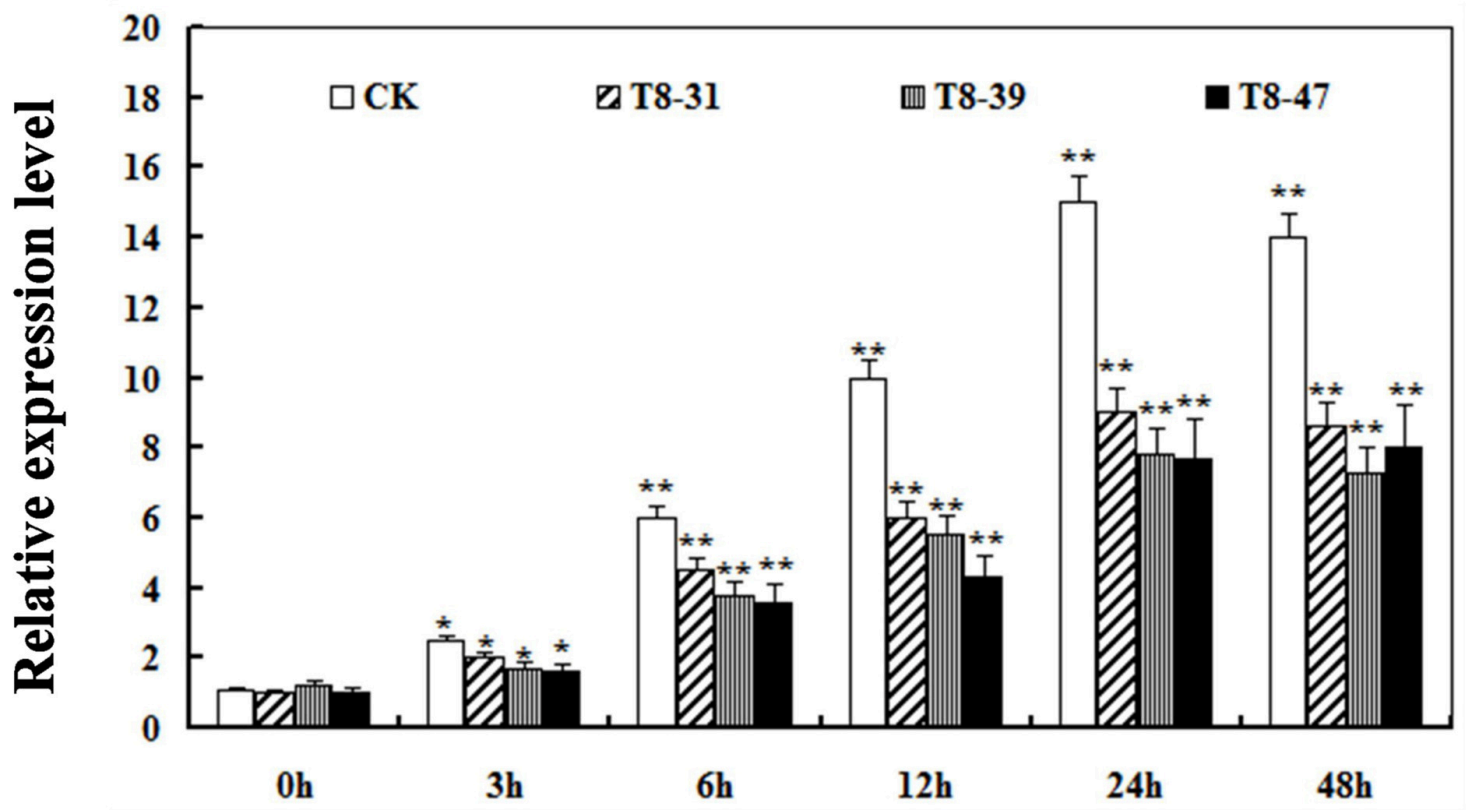
**Relative expression levels of reactive oxygen species (ROS) in transgenic soybean plants and non-transgenic soybean plants at 0, 3, 6, 12, 24, 48 h after *P. sojae* infection**. Values are relative to the value of mock plants at the same time point. Statistically significant differences were performed between the overexpression transgenic lines and non-transgenic lines. Three biological replicates with their three technical replicates were averaged and statistically analyzed using Student's *t*-test (^*^P < 0.05; ^**^P < 0.01). Bars indicate standard error of the mean.

It is known that pathogen infection is associated with the production of ROS (Hückelhoven and Kogel, 2003; Soosaar et al., 2005; Takabatake et al., 2007; Shetty et al., 2008). Therefore, the ROS relative expression levels was detected in three T8 transgenic soybean plants (T8-31, T8-39, and T8-47) and non-transgenic plants at 0, 3, 6, 12, 24, 48 h after incubation with *P. sojae*. The results showed that the relative expression levels of ROS gradually increased in transgenic plants and non-transgenic plants with the incubation period (Figure 6). The relative expression levels of ROS in the transgenic plants were significantly lower than those of non-transgenic plants at the same time point (Figure 6), suggesting that an important role of *GmIFR* might function as an antioxidant to reduce ROS in soybean.

### Overexpression of GmIFR in soybean seed affects isoflavone and glyceollins expression levels

**Figure 7:**
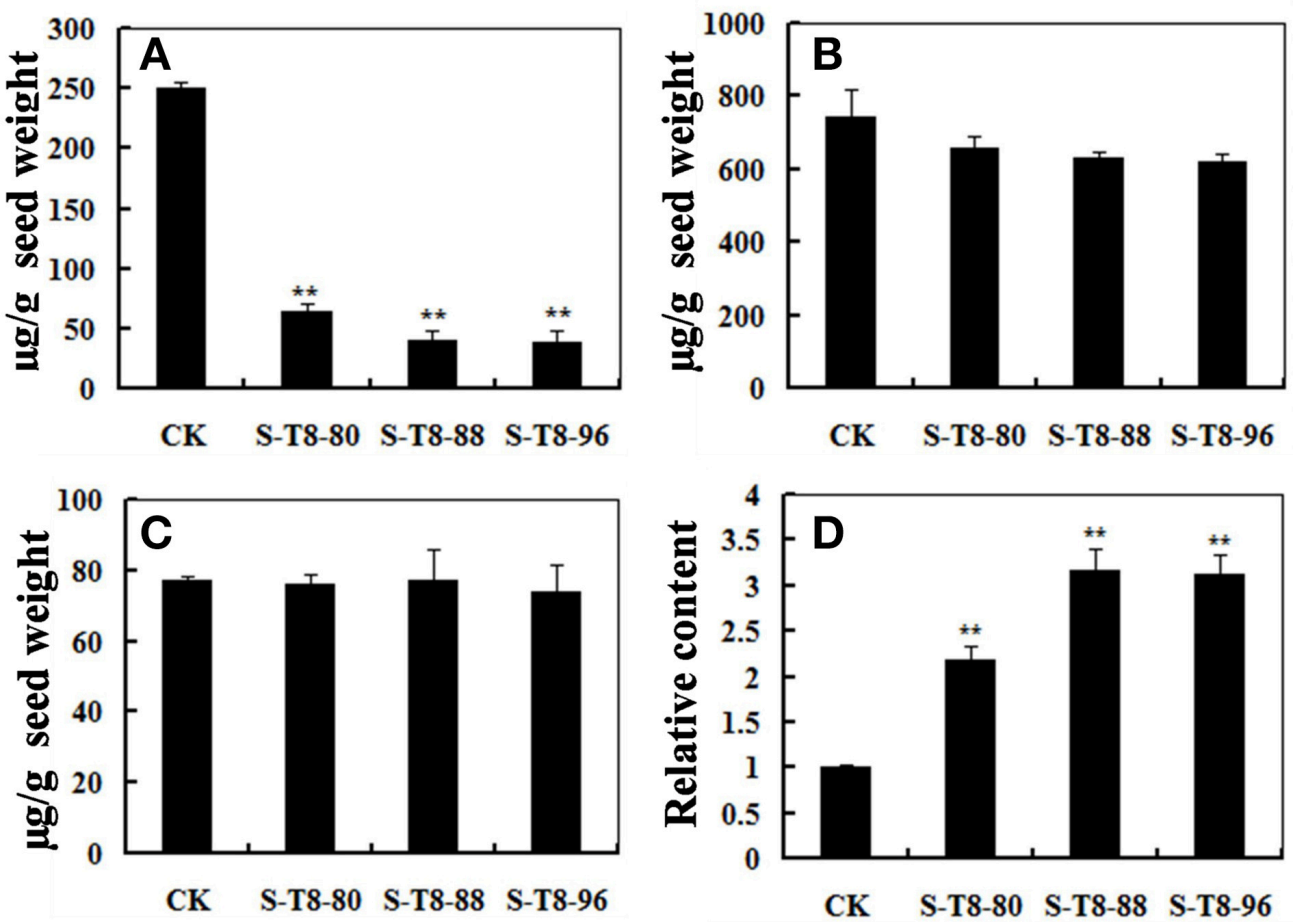
**The content of isoflavone components and the relative content of glyceollins in seeds of transgenic and non-transgenic soybeans**. **(A)** The daidzein levels in seeds of transgenic and non-transgenic soybeans. **(B)** The glycitein levels in seeds of transgenic and non-transgenic soybeans. **(C)** The genistein levels in seeds of transgenic and non-transgenic soybeans. **(D)** The relative content of glyceollins in the seeds of transgenic and non-transgenic soybeans. The experiment was performed three biological replicates with their respective three technical replicates and statistically analyzed using Student's *t*-test (^**^P < 0.01). Bars indicate standard error of the mean.

It is well known that daidzein, genistein, and glycitein were the essential components of isoflavones (Wang et al., 2014) and IFR was a crucial enzyme involved in the synthesis of the glyceollins from daidzein (Graham et al., 1990; Oliver et al., 2003). Thus, changes in the *IFR* expression level may cause change of isoflavonoid and glyceollins content in soybean. To study the relationship between isoflavonoid and glyceollins content in soybean seeds and the *GmIFR* gene expression level, the isoflavonoid content and the relative content of the glyceollins were measured in the seeds of transgenic soybean plants and non-transgenic soybean plants. As shown in Figure 7A, the daidzein levels in the seeds (numbered S-T8-80, S-T8-88, and S-T8-96) developed from the three independent transformed lines T8-80, T8-88, and T8-96 reduced greatly, while levels of genistein and glycitein had little change compared to those of control (Figures 7B,C). The relative content of glyceollins in the transgenic plants was significantly higher than that of non-transgenic plants (Figure 7D), suggesting that an important role of *GmIFR* involved in the synthesis of the glyceollins from daidzein in soybean.

### Over-expressing of *GmIFR* affected the transcriptional level of multiple genes involved in phenylpropanal pathway

**Figure 8:**
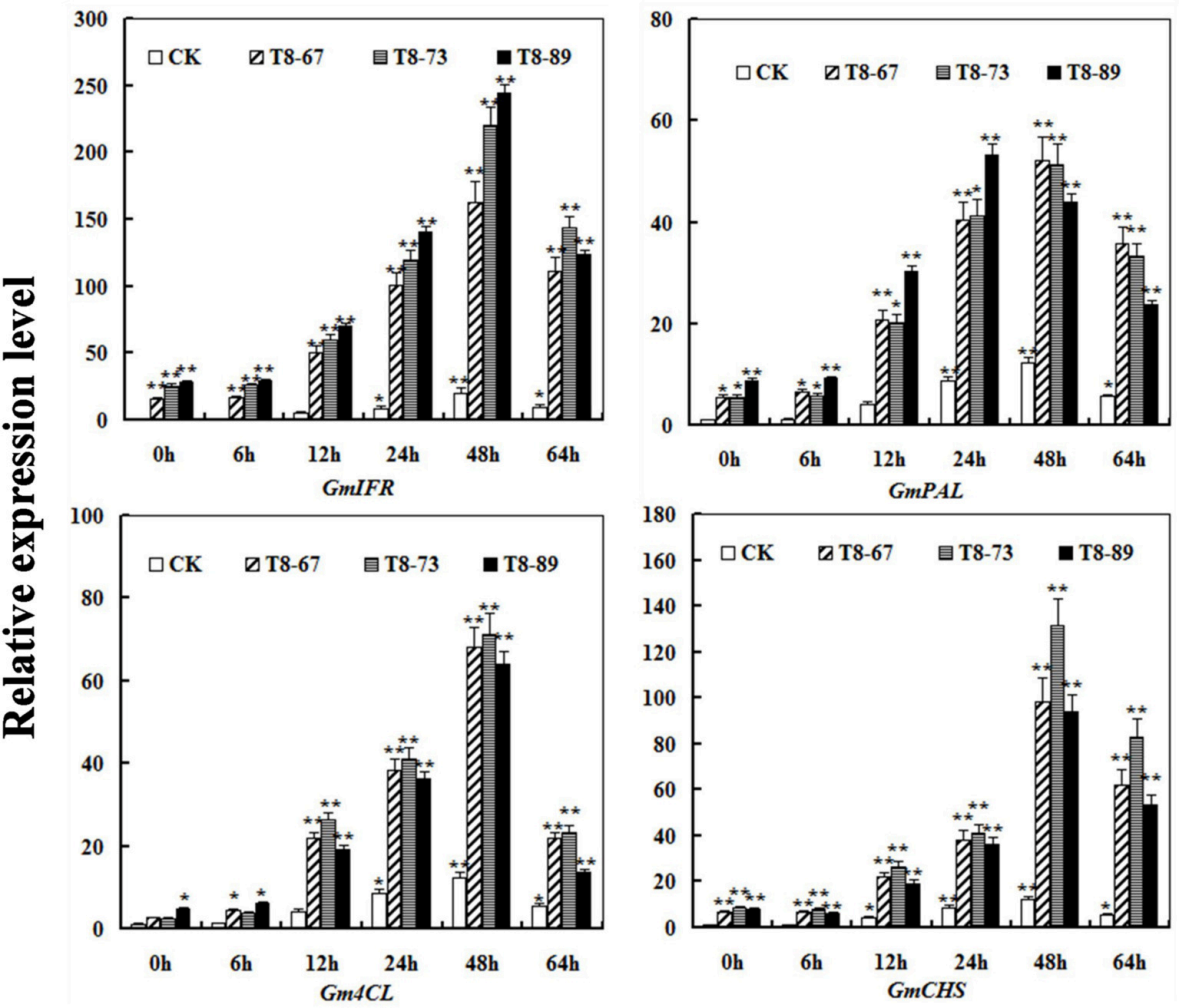
**The transcript levels of the three genes (*GmPAL, Gm4CL, GmCHS*) in *GmIFR* transgenic and non-transgenic soybean plants after *P. sojae* infection using quantitative RT-PCR analysis**. The amplification of the soybean *Actin* (*GmActin4*) gene was used as an internal control to normalize all the data. The relative transcript levels of the genes were quantified compared with mock plants at the same time point. The experiment was performed on three biological replicates with their respective three technical replicates and statistically analyzed using Student's *t*-test (^*^P < 0.05; ^**^P < 0.01). Bars indicate standard error of the mean.

To test whether GmIFR protein could regulate stress-related genes expression in the isoflavonoids synthesis pathway, the expression of *GmIFR* and three stress-related genes (*GmPAL*,*Gm4CL, GmCHS*) was analyzed in transgenic soybean plants and non-transgenic plants using qRT-PCR at 0, 6, 12, 24, 48, 64 h after incubation with *P. sojae*. As shown in Figure 8, the transcript levels of *GmPAL*,*Gm4CL, GmCHS* were induced after incubation with *P. sojae* in transgenic soybean plants and non-transgenic soybean, and the transcript levels of the three genes were significantly higher than those of non-transgenic soybean plants at the same time point.

## Discussion

In this study, we identified a novel *GmIFR* gene that encodes for a NAD(P)H -dependant oxidoreductase, enhances resistance to *P. sojae* when over-expressed in soybean. The gene encoding isoflavonoid reductase (IFR), one of the key enzymes in isoflavonoid phytoalexin biosynthesis, was first cloned from alfalfa (*Medicago sativa* L.; Paiva et al., 1991). However, there is little knowledge about the biological function of IFR in soybean. Here, we report for the first time that *GmIFR* transgenic soybean plants inoculated with *P. sojae* display significantly altered responses to pathogen infection.

Plants encounter a range of environmental stresses in their natural environments and have evolved a wide range of mechanisms to cope with them (Dixon and Paiva, 1995; Zhang et al., 2008). There are multiple stress perception and signaling pathways, some of which are specific, whereas others cross-talk at various steps (Kunkel and Brooks, 2002; Chinnusamy et al., 2004; Fujita et al., 2006; Rípodas et al., 2013). It has been reported that higher levels of IFR determining fungal resistance in response to *A. rabiei* in chickpea (Daniel et al., 1990). In the present study, we demonstrated that overexpression of the *GmIFR* gene improved resistance to *P. sojae* in soybean. Moreover, the genes with high sequence similarity to IFRs have been identified in non-leguminous plants and are called isoflavone reductases like (IRL; Shoji et al., 2002). In most cases, IRLs might involve in responses to biotic or abiotic stresses (Shoji et al., 2002; Zhu et al., 2009; Cheng et al., 2013). A recent study suggested that GbIRL is involved in regulating ABA, JA, and ET stress responses in Ginkgo (Cheng et al., 2013). In this study, the expression of *GmIFR* following various stress treatments was analyzed. The results showed that inoculation with *P. sojae* as biotic stress and woundings as abiotic stress significantly increased the transcript levels of *GmIFR* in soybean plants. The expression of *GmIFR* was also induced by treatments with SA, ET, JA, and ABA. ABA is a phytohormone that is extensively involved in responses to abiotic stresses such as drought and low temperature, as well as osmotic stress (Skriver and Mundy, 1990). In contrast, the phytohormones SA, JA, and ET play central roles in biotic stress signaling following pathogen infection (Pieterse et al., 2009; Robert-Seilaniantz et al., 2011; Sugano et al., 2013). Therefore, it is possible to speculate that *GmIFR* likely plays an important role in responsive to biotic stresses in soybean. Moreover, it has been reported that IFR proteins as the key enzymes can catalyze reductase reactions (Paiva et al., 1991; Gang et al., 1999). For instance, IFR can convert 2′-hydroxyformononetin of the isoflavonoid substrates to (3R)-vestitone in alfalfa (Paiva et al., 1991). In Ginkgo, the recombinant GbIRL1 protein could catalyze the formation of the TDDC, IDDDC from DDDC, DDC (Cheng et al., 2013). In this work, we determined the recombinant GmIFR protein could catalyze oxidoreductase reaction using enzyme assays. Therefore, it is proven that GmIFR has isoflavone reductase activity.

There have been some reports about the inhibition of glyceollin on several lines of pathogen species (Lyne et al., 1976; Kim et al., 2010a, 2012; Nwachukwu et al., 2013). More specifically, the glyceollin can resist to *Phytophthora megasperma* var. *sojae* in soybean (Lyne et al., 1976; Hahn et al., 1985; Lygin et al., 2010, 2013). In addition, Boue and Raina (2003) noted that the fungi *Aspergillus flavus, Aspergillus niger, Aspergillus oryzae*, and *Aspergillus flavus* were all able to induce glyceollin in soybean. Glyceollin revealed a remarkable antimicrobial effect against *Phytophthora capsici* and *Sclerotinia scrotiorum* by Kim et al. (2010a). Moreover, It has been reported that IFR was a key enzyme involved in the synthesis of the glyceollins from daidzein which was the essential component of the isoflavones (Graham et al., 1990; Oliver et al., 2003). In this work, we detected the content of daidzein, genistein, glycitein and the relative content of glyceollins in transgenic soybean seeds and non-transgenic soybean seeds. The results showed that the daidzein content greatly reduced in transgenic soybean seeds, while levels of genistein and glycitein had little change compared to those of the non-transgenic soybean seeds. The relative content of glyceollins in the transgenic plants was significantly higher than that of non-transgenic plants. Therefore, we suggested that *GmIFR* might improve the resistance to *P. sojae* in soybean when overexpression likely by increasing the accumulation of glyceollins. It has been reported that the biosynthesis of glyceollin via the isoflavonoid branch of the phenylpropanoid pathway (Ng et al., 2011) and glyceollin as a phytoalexin could response to pathogen invasion (Hahn et al., 1985; Lygin et al., 2010; Kim et al., 2012). PAL, 4CL, and CHS play an important role in the isoflavonoid branch of the phenylpropanoid pathway (Vogt, 2010; Yi et al., 2010). Here, we studied the transcript levels change of the three genes (*GmPAL, GmCHS, Gm4CL*) after incubation with *P. sojae* in transgenic and non-transgenic soybean plants. We found that these genes were induced after incubation with *P. sojae* and there was up-regulation of the transcript in transgenic soybean plants. We speculated that the three genes (*GmPAL, GmCHS, Gm4CL*) might play a cooperation role on the biosynthesis of glyceollin which might improve resistance to *P. sojae* in soybean.

Plants experience a variety of environmental stresses likely leading to the generation of ROS (Sies, 1991). Although ROS may be essential to maintain homoeostasis, the generation of ROS, within certain boundaries, is harmful at the high concentration (Kim et al., 2012). However, glyceollins as major isoflavonoid phytoalexin in leguminous plants, showed strong antioxidant activity and ROS scavenging potential when assessed by an *in vitro* model (Kim et al., 2010a, 2012). Moreover, Kim et al. (2010b) reported that overexpression of *OsIRL* in transgenic rice plants promotes resistance to ROS. A possible interpretation is that the IRLs contain a putative NAD (P) domain related to oxidation/reduction properties (Babiychuk et al., 1995; Petrucco et al., 1996). The different roles of ROS in plants interactions with special emphasis on fungal and oomycete pathogens have also been reported (Shetty et al., 2008). For example, *Blumeria graminis* f. sp. *hordei* can induce ROS accumulation in barley (Hückelhoven and Kogel, 2003) and *Septoria tritici* can induce ROS accumulation in wheat (Shetty et al., 2003). Thus, it can be deduced that the plants can improve resistance to pathogen by scavenge excess ROS. Consistent with these, we detected the relative expression levels of ROS in transgenic soybean plants and non-transgenic plants after incubation with *P. sojae*, and found that the relative expression levels of ROS in transgenic plants were significantly lower than those of control plants. Overexpression of *GmIFR* in transgenic soybean plants can increase the content of glyceollins or GmIFR itself behave as an antioxidant to scavenge ROS, which might improve resistance to *P. sojae* in soybean.

### Conflict of interest statement

The authors declare that the research was conducted in the absence of any commercial or financial relationships that could be construed as a potential conflict of interest.

## Supplementary material

Supplementary Table S1 **Oligonucleotide primers used in this study**.

Supplementary Figure S1 **The nucleotide sequence of GmIFR cDNA together with its predicted amino acid sequence**. The NAD (P) domain is underlined.

Supplementary Figure S2 **Phylogenetic analysis and sequence alignment of GmIFR in the leguminous plants genome. (A)** The predicted three-dimensional model of the IFR. **(B)** Phylogenetic relationships of GmIFR with IFRs proteins; the phylogenetic tree was constructed using amino acid sequences of the IFRs protein from other species. The plant species and GenBank accession numbers were as follows: *Glycine max* GmIFR (NM_001254100); *Phaseolus vulgaris* PvIFR (XP_007156276.1), *Cicer arietinum* CaIFR (XP_004509553.1), *Medicago truncatula* MtIRF (AFK37791.1), *Lotus japonicus* LjIFR (BAF34845.1), *Glycine soja* GsIFR (KHN36092.1), *Medicago sativa* MsIFR (CAA41106), *Pisum sativum* PsIFR (P52576.1). **(C)** The conserved NAD (P) domain of the IFR group proteins.

## References

[B1] Babiychuk E. Kushnir S. Belles-Boix E. Van Montagu M. Inz'e D. (1995). *Arabidposis thaliana* NADPH oxidoreductase homologs confer tolerance of yeast toward the thiol-oxidizing drug diamide. J. Biol. Chem. 270, 26224–26231. 10.1074/jbc.270.44.26224 7592828

[B2] Banks S. W. Dewick P. M. (1983). Biosynthesis of glyceollins I, II and III in soybean. Phytochemistry 22, 2729–2733. 10.1016/S0031-9422(00)97682-9

[B3] Boue S. M. Carter C. H. Ehrlich K. C. Clevel T. E. (2000). Induction of the soybean phytoalexins coumestrol and glyceollin by *Aspergillus*. J. Agric. Food Chem. 48, 2167–2172. 10.1021/jf9912809 10888516

[B4] Boue S. M. Raina A. K. (2003). Effects of plant flavonoids on fecundity, survival, and feeding of the Formosan subterranean termite. J. Chem. Ecol. 29, 2575–2584. 10.1023/A:1026318203775 14682534

[B5] Cheng H. Li L. L. Xu F. Wang Y. Yuan H. H. Wu C. H.. (2013). Expression patterns of an isoflavone reductase-like gene and its possible roles in secondary metabolism in *Ginkgo biloba*. Plant Cell Rep. 32, 637–650. 10.1007/s00299-013-1397-2 23459862

[B6] Chinnusamy V. Schumaker K. Zhu J. K. (2004). Molecular genetic perspectives on cross-talk and specificity in abiotic stress signalling in plants. J. Exp. Bot. 55, 225–236. 10.1093/jxb/erh005 14673035

[B7] Cooper J. D. Qiu F. Paiva N. L. (2002). Biotransformation of an exogenously supplied isoflavonoid by transgenic tobacco cells expressing alfalfa isoflavone reductase. Plant Cell Rep. 20, 876–884. 10.1007/s00299-001-0404-1

[B8] Daniel S. Tiemann K. Wittkampf U. Bless W. Hinderer W. Barz W. (1990). Elicitor-induced metabolic changes in cell cultures of chickpea *(Cicer arietinum* L.) cultivars resistant and susceptible to *Ascochyta rabiei*. Planta 182, 270–278. 24197106 10.1007/BF00197121

[B9] Dixon R. A. Paiva N. L. (1995). Stress-induced phenylpropanoid metabolism. Plant Cell 7, 1085–1097. 10.1105/tpc.7.7.1085 12242399 PMC160915

[B10] Dou D. L. Wang B. S. Zhu S. W. Tang Y. X. Wang Z. X. Sun J. S. (2003). Transgenic tobacco with NDR1 gene improved its resistance to two fungal disease. Sci. Agric. Sin. 36, 1120–1124.

[B11] Fehr W. R. Caviness C. E. Burmood D. T. Pennington J. (1971). Stage of development descriptions for soybeans, *Glycine max* (L.) Merrill. Crop Sci. 11, 929–931. 10.2135/cropsci1971.0011183X001100060051x

[B12] Fischer D. Ebeanau-Jehle C. Grisebach H. (1990). Phytoalexin synthesis in soybean: purification and characterization of NADPH: 20-hydroxydaidzein oxidoreductase from elicitor-challenged soybean cellcultures. Arch. Biochem. Biophys. 276, 390–395. 10.1016/0003-9861(90)90737-J 2306102

[B13] Fujita M. Fujita Y. Noutoshi Y. Takahashi F. Narusaka Y. Yamaguchi-Shinozaki K.. (2006). Crosstalk between abiotic and biotic stress responses: a current view from the points of convergence in the stress signaling networks. Curr. Opin. Plant Biol. 9, 436–442. 10.1016/j.pbi.2006.05.014 16759898

[B14] Gang D. R. Kasahara H. Xia Z. Q. Vander Mijnsbrugge K. Bauw G. Boerjan W.. (1999). Evolution of plant defense mechanisms: relationships of phenylcoumaran benzylic ether reductases to pinoresinol lariciresinol and isoflavone reductases. J. Biol. Chem. 274, 7516–7527. 10.1074/jbc.274.11.7516 10066819

[B15] Graham T. L. Kim J. E. Graham M. Y. (1990). Role of constitutive isoflavone conjugates in the accumulation of glyceollin in soybean infected with *Phytophthora megasperma*. Mol. Plant Microbe 3, 157–166. 10.1094/MPMI-3-157

[B16] Guo L. Dixon R. A. Paiva N. L. (1994). Conversion of vestitone to medicarpin in alfalfa (*Medicago sativa* L.) is catalyzed by two independent enzymes. J. Biol. Chem. 269, 22372–22378. 8071365

[B17] Hahn M. G. Bonhoff A. Grisebach H. (1985). Quantitative localization of the phytoalexin glyceollinI in relation to fungal hyphae in soybean roots infected with *Phytophthora megasperma* f. sp. *glycinea*. Plant Physiol. 77, 591–601. 10.1104/pp.77.3.591 16664104 PMC1064570

[B18] Hückelhoven R. Kogel K. H. (2003). Reactive oxygen intermediates in plant microbe interactions: who is who in powdery mildew resistance? Planta 216, 891–902. 10.1007/s00425-003-0973-z 12687357

[B19] Kim H. J. Lim J. S. Kim W. K. Kim J. S. (2012). Soybean glyceollins: biological effects and relevance to human health. Proc. Nutr. Soc. 71, 166–174. 10.1017/S0029665111003272 22054259

[B20] Kim H. J. Suh H. J. Kim J. H. Park S. Joo Y. C. Kim J. S. (2010a). Antioxidant activity of glyceollins derived from soybean elicited with *Aspergillus sojae*. Agric. Food Chem. 58, 11633–11638. 10.1021/jf102829z 21033668

[B21] Kim S. G. Kim S. T. Wang Y. M. Kim S. K. Lee C. H. Kim K. K.. (2010b). Overexpression of rice isoflavone reductase-like gene (*OsIRL*) confers tolerance to reactive oxygen species. Physiol. Plant 138, 1–9. 10.1111/j.1399-3054.2009.01290.x 19825006

[B22] Kim S. T. Cho K. S. Kim S. G. Kang S. Y. Kang K. Y. (2003). A rice isoflavone reductase-like gene, *OsIRL*, is induced by rice blast fungal elicitor. Mol. Cells 16, 224–231. 14651265

[B23] Kunkel B. N. Brooks D. M. (2002). Cross talk between signaling pathways in pathogen defense. Curr. Opin. Plant Biol. 5, 325–331. 10.1016/S1369-5266(02)00275-3 12179966

[B24] Lers A. Bud S. Lomanic E. Droby S. Chalutz E. (1998). The expression of a grapefruit gene encoding an isoflavone reductase-like protein is induced in response to UV-irradiation. Plant Mol. Biol. 36, 847–856. 10.1023/A:1005996515602 9520276

[B25] Lin W. (1983). Isolation of mesophyll protoplasts from mature leaves of soybeans. Plant Physiol. 73, 1067–1069. 10.1104/pp.73.4.1067 16663331 PMC1066609

[B26] Lygin A. V. Hill C. B. Zernova O. V. Crull L. Widholm J. M. Hartman G. L.. (2010). Response of soybean pathogens to glyceollin. Phytopathology 100, 897–903. 10.1094/PHYTO-100-9-0897 20701487

[B27] Lygin A. V. Zernova O. V. Hill C. B. Kholina N. A. Widholm J. M. Hartman G. L.. (2013). Glyceollin is an important component of soybean plant defense against *Phytophthora sojae* and *Macrophomina phaseolina*. Phytopathology 103, 984–994. 10.1094/PHYTO-12-12-0328-R 23617338

[B28] Lyne R. L. Mulheirn L. J. Leworthy D. P. (1976). New pterocarpinoid phytoalexins of soybean. J. Chem. Soc. Chem. Commun. 13, 497–498. 10.1039/c39760000497

[B29] Morris P. F. Savard M. E. Ward E. W. B. (1991). Identification and accumulation of isoflavonoids and isoflavone glucosides in soybean leaves and hypocotyls in resistance responses to *Phytophthora megasperma* f. sp. glycines. Physiol. Mol. Plant 39, 229–224. 10.1016/0885-5765(91)90006-4

[B30] Morrison R. H. Thorne J. C. (1978). Inoculation of detached cotyledons for screening soybeans against two races of *Phytophthora megasperma* var. *sojae*. Crop Sci. 18, 1089–1091. 10.2135/cropsci1978.0011183X001800060049x

[B31] Ng T. B. Ye X. J. Wong J. H. Fang E. F. Chan Y. S. Pan W. L.. (2011). Glyceollin, a soybean phytoalexin with medicinal properties. Appl. Microbiol. Biotechnol. 90, 59–68. 10.1007/s00253-011-3169-7 21336922

[B32] Nwachukwu I. D. Luciano F. B. Udenigwe C. C. (2013). The inducible soybean glyceollin phytoalexins with multifunctional health-promoting properties. Food Res. Int. 54, 1208–1216. 10.1016/j.foodres.2013.01.024

[B33] Oliver Y. Shi J. Hession A. O. Maxwell C. A. McGongigle B. Odell J. T. (2003). Metabolic engineering to increase isoflavone biosynthesis in soybean seed. Phytochemistry 63, 753–763. 10.1016/S0031-9422(03)00345-5 12877915

[B34] Paiva N. L. Edwards R. Sun Y. J. Hrazdina G. Dixon R. A. (1991). Stress responses in alfalfa (*Medicago sativa* L.) 11. Molecular cloning and expression of alfalfa isoflavone reductase, a key enzyme of isoflavonoid phytoalexin biosynthesis. Plant Mol. Biol. 17, 653–667. 10.1007/BF00037051 1912490

[B35] Partridge J. E. Keen N. T. (1977). Soybean phytoalexins: rates of synthesis are not regulated by activation of initial enzymes in flavonoid biosynthesis. J. Physiol. Biochem. 67, 50–55. 10.1094/phyto-67-50

[B36] Paz M. M. Shou H. X. Guo Z. B. Zhang Z. Y. Banerjee A. K. Wang K. (2004). Assessment of conditions affecting *Agrobacterium*-mediated soybean transformation using the cotyledonary node explant. Euphytica 136, 167–179. 10.1023/B:EUPH.0000030669.75809.dc

[B37] Petrucco S. Bolchi A. Foroni C. Percudani R. Rossi G. L. Ottonello S. (1996). A maize gene encoding an NADPH binding enzyme highly homologous to isoflavone reductases is activated in response to sulfur starvation. Plant Cell 8, 69–80. 10.1105/tpc.8.1.69 8597660 PMC161082

[B38] Pieterse C. M. J. Leon-Reye A. Van der Ent S. Van Wees S. C.. (2009). Networking by smallmolecule hormones in plant immunity. Nat. Chem. Biol. 5, 308–316. 10.1038/nchembio.164 19377457

[B39] Qian H. F. Chen W. Sun L. W. Jin Y. X. Liu W. P. Fu Z. W. (2009). Inhibitory effects of paraquat on photosynthesis and the response to oxidative stress in *Chlorella vulgaris*. Ecotoxicology 18, 537–543. 10.1007/s10646-009-0311-8 19377883

[B40] Rípodas C. Via V. D. Aguilar O. D. Zanetti M. E. Blanco F. A. (2013). Knock-down of a member of the isoflavone reductase gene family impairs plant growth and nodulation in *Phaseolus vulgaris*. Plant Physiol. Bioch. 68, 81–89. 10.1016/j.plaphy.2013.04.003 23644278

[B41] Robert-Seilaniantz A. Grant M. Jones J. D. G. (2011). Hormone crosstalk in plant disease and defense: more than just jasmonate-salicy late antagonism. Annu. Rev. Phytopathol. 49, 317–343. 10.1146/annurev-phyto-073009-114447 21663438

[B42] Shetty N. P. Kristensen B. K. Newman M. A. Møller K. Gregersen P. L. Jørgensen H. J. L. (2003). Association of hydrogen peroxide with restriction of Septoria tritici in resistant wheat. Physiol. Mol. Plant 62, 333–346. 10.1016/S0885-5765(03)00079-1

[B43] Shetty N. P. Lyngs Jørgensen H. J. Jensen J. D. Collinge D. B. Shetty H. S. (2008). Roles of reactive oxygen species in interactions between plants and pathogens. Eur. J. Plant Pathol. 121, 267–280. 10.1007/s10658-008-9302-5

[B44] Shoji T. Winz R. Iwase T. Nakajima K. Yamada Y. Hashimoto T. (2002). Expression patterns of two tobacco isoflavone reductase-like genes and their possible roles in secondary metabolism in tobacco. Plant Mol. Biol. 50, 427–440. 10.1023/A:1019867732278 12369619

[B45] Sies H. (1991). Oxidative stress: from basic research to clinical application. Am. J. Med. 91, 31–38. 10.1016/0002-9343(91)90281-2 1928209

[B46] Skriver K. Mundy J. (1990). Gene expression in response to abscisic acid and osmotic stress. Plant Cell 2:503. 10.1105/tpc.2.6.503 2152172 PMC159906

[B47] Somerville C. Somerville S. (1999). Plant functional genomics. Science 285, 380–383. 10.1126/science.285.5426.380 10411495

[B48] Soosaar J. L. M. Burch-Smith T. M. Dinesh-Kumar S. P. (2005). Mechanisms of plant resistance to viruses. Nat. Rev. Microbiol. 3, 789–798. 10.1038/nrmicro1239 16132037

[B49] Sugano S. Sugimoto T. Takatsuji H. Jiang J. (2013). Induction of resistance to *Phytophthora sojae* in soybean (*Glycine max*) by salicylic acid and ethylene. Plant Pathol. 62, 1048–1056. 10.1111/ppa.12011

[B50] Sun Y. J. Wu Q. D. VanEtten H. D. Hrazdina G. (1991). Stereoisomerism in plant disease resistance: induction and isolation of the 7, 20-dihydroxy-40, 50-methylenedioxyisoflavone reductase, an enzyme introducing chirality during synthesis of isoflavanoid phytoalexins in pea (*Pisum sativum* L). Arch. Biochem. Biophys. 284, 167–173. 10.1016/0003-9861(91)90279-R 1989493

[B51] Takabatake R. Ando Y. Seo S. Katou S. Tsuda S. Ohashi Y.. (2007). MAP kinases function downstream of HSP90 and upstream of mitochondria in TMV resistance gene *N*-mediated hypersensitive cell death. Plant Cell Physiol. 48, 498–510. 10.1093/pcp/pcm021 17289794

[B52] Tamura K. Dudley J. Nei M. Kumar S. (2007). MEGA4: molecular evolutionary genetics analysis (MEGA) software version 4.0. Mol. Biol. Evol. 24, 1596–1599. 10.1093/molbev/msm092 17488738

[B53] Vogt T. (2010). Phenylpropanoid biosynthesis. Mol. Plant 3, 2–20. 10.1093/mp/ssp106 20035037

[B54] Wang X. Q. He X. Z. Lin J. Q. Shao H. Chang Z. Z. Dixon R. A. (2006). Crystal structure of isoflavone reductase from alfalfa (*Medicago sativa* L.). J. Mol. Biol. 358, 1341–1352. 10.1016/j.jmb.2006.03.022 16600295

[B55] Wang Y. Han Y. P. Teng W. L. Zhao X. Li Y. G. Wu L.. (2014). Expression quantitative trait loci infer the regulation of isoflavone accumulation in soybean (*Glycine max* L. *Merr.)* seed. BMC Genomics 15:680. 10.1186/1471-2164-15-680 25124843 PMC4138391

[B56] Ward E. W. B. Lazarovits G. Unwin C. H. Buzzell R. I. (1979). Hypocotyl reactions and glyceollin in soybeans inoculated with zoospores of *Phytophthora megaspuma* var. sojae. Phytopathology 69, 951–955. 10.1094/Phyto-69-951

[B57] Xu P. F. Chen W. Y. Lv H. Y. Fan S. J. Wang X. Jiang L. Y. (2012). Differentially expressed genes of soybean during infection by *Phytophthora sojae*. J. Integr. Agric. 11, 368–377. 10.1016/S2095-3119(12)60021-5

[B58] Yi J. X. Derynck M. R. Li X. Y. Telmer P. Marsolais F. Dhaubhadel S. (2010). A single-repeat MYB transcription factor, GmMYB176, regulates CHS8 gene expression and affects isoflavonoid biosynthesis in soybean. Plant J. 62, 1019–1034. 10.1111/j.1365-313x.2010.04214.x 20345602

[B59] Yoo S. D. Cho Y. H. Sheen J. (2007). Arabidopsis mesophyll protoplasts: a versatile cell system for transient gene expression analysis. Nat. Protoc. 2, 1565–1572. 10.1038/nprot.2007.199 17585298

[B60] Yoshikawa M. Yamauchi K. Masago H. (1978). Glyceollin: its role in restricting fungal growth in resistant soybean hypocotyls infected with *Phytophthora megasperma* var. *sojae*. Physiol. Mol. Plant 12, 73–82. 10.1016/0048-4059(78)90020-6

[B61] Zeng G. L. Li D. M. Han Y. P. Teng W. L. Wang J. Qiu L. Q.. (2009). Identification of QTL underlying isoflavone contents in soybean seeds among multiple environments. Theo. Appl. Genet. 118, 1455–1463. 10.1007/s00122-009-0994-5 19266178

[B62] Zhang G. Y. Chen M. Chen X. P. Xu Z. S. Guan S. Li L. C.. (2008). Phylogeny, gene structures, and expression patterns of the ERF gene family in soybean (*Glycine max* L.). J. Exp. Bot. 59, 4095–4107. 10.1093/jxb/ern248 18832187 PMC2639015

[B63] Zhang S. Z. Xu P. F. Wu J. J. Allen X. Zhang J. X. Li W. B. (2010). Races of *Phytophthora sojae* and their virulences on commonly grown soybean varieties in Heilongjiang, China. Plant Dis. 94, 87–91. 10.1094/PDIS-94-1-0087 30754393

[B64] Zhu Q. Guo T. Sui S. Liu G. Lei X. Luo L.. (2009). Molecular cloning and characterization of a novel isoflavone reductase-like gene (*FcIRL*) from high flavonoids-producing callus of *Fagopyrum cymosum*. Acta. Pharmacol. Sin. 44, 809–819. 19806925

