# Supplementary material for: Overexpression of Soybean Isoflavone Reductase (GmIFR) Enhances Resistance to Phytophthora sojae in Soybean
Source: Front Plant Sci. 2015 Nov 23;6:1024. doi: 10.3389/fpls.2015.01024 (PMC4655237; doi:10.3389/fpls.2015.01024)
Supplement: Supplementary Table S1 — Oligonucleotide primers used in this study. [file Table1.DOC]

**Supplemental Table 1.** Oligonucleotide primers used in this study.

| **Gene cloning** | *GmIFRF* | GGGGTATTGAGATTGAGATTC |
| --- | --- | --- |
|  | *GmIFRR* | AACCGTGTTCCTGTTTGA |
|  | *GmIFRTF* | GCTCTAGAGGGGTATTGAGATTGAGATTC |
|  | *GmIFRTR* | CGAGCTCAACCGTGTTCCTGTTTGA |
| **qRT-PCR** | *GmIFRQF* | GAGGAGGATAGTGAGGGCAA |
|  | *GmIFRQR* | CCTGATGTGAACACCAGAGATG |
|  | *GmActin4F* | GTGTCAGCCATACTGTCCCCATTT |
|  | *GmActin4R* | GTTTCAAGCTCTTGCTCGTAATCA |
|  | *EF1F* | CCACTGCTGAAGAAGATGATGATG |
|  | *EF1R* | AAGGACAGAAGACTTGCCACTC |
|  | *GmPALF* | TAACTGGCAGACCCAACTCC |
|  | *GmPALR* | CCATTAACAAGGGCAAGACC |
|  | *Gm4CLF* | ATTCTATTCTCCTCTGGCACCA |
|  | *Gm4CLR* | CCATACCTCTCCACAGCCTTTA |
|  | *GmCHSF* | AGTCGTAGTAGACGGTGGAAAAA |
|  | *GmCHSR* | AATCAGAGAGGTTGAAATGAAGG |
| **Yeast two-hybrid** | *GmIFRYF* | ATGGAGGCCGAATTCGTAATGGGGAAGAGCAAGGT |
|  | *GmIFRYR* | AATTCCCGGGGATCCACACATAAATTTTTAGGTACTCG |
| **GFP** | *GmIFRGF* | GCCCATGGGGGGTATTGAGATTGAGATTC |
|  | *GmIFRGR* | CACTAGTAACCGTGTTCCTGTTTGA |
| **Enzyme assays** | *GmIFRPF* | CCATGGTAATGGGGAAGAGCAAGGT |
|  | *GmIFRPR* | CTCGAGCACATAAATTTTTAGGTAC |
