# Supplementary figures and images for: Overexpression of Soybean Isoflavone Reductase (GmIFR) Enhances Resistance to Phytophthora sojae in Soybean
Source: Front Plant Sci. 2015 Nov 23;6:1024. doi: 10.3389/fpls.2015.01024 (PMC4655237; doi:10.3389/fpls.2015.01024)

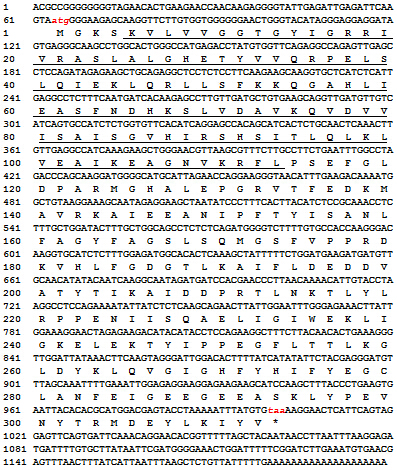

Supplement: Supplementary Figure S1 — The nucleotide sequence of GmIFR cDNA together with its predicted amino acid sequence. The NAD (P) domain is underlined. [file Image1.JPEG]

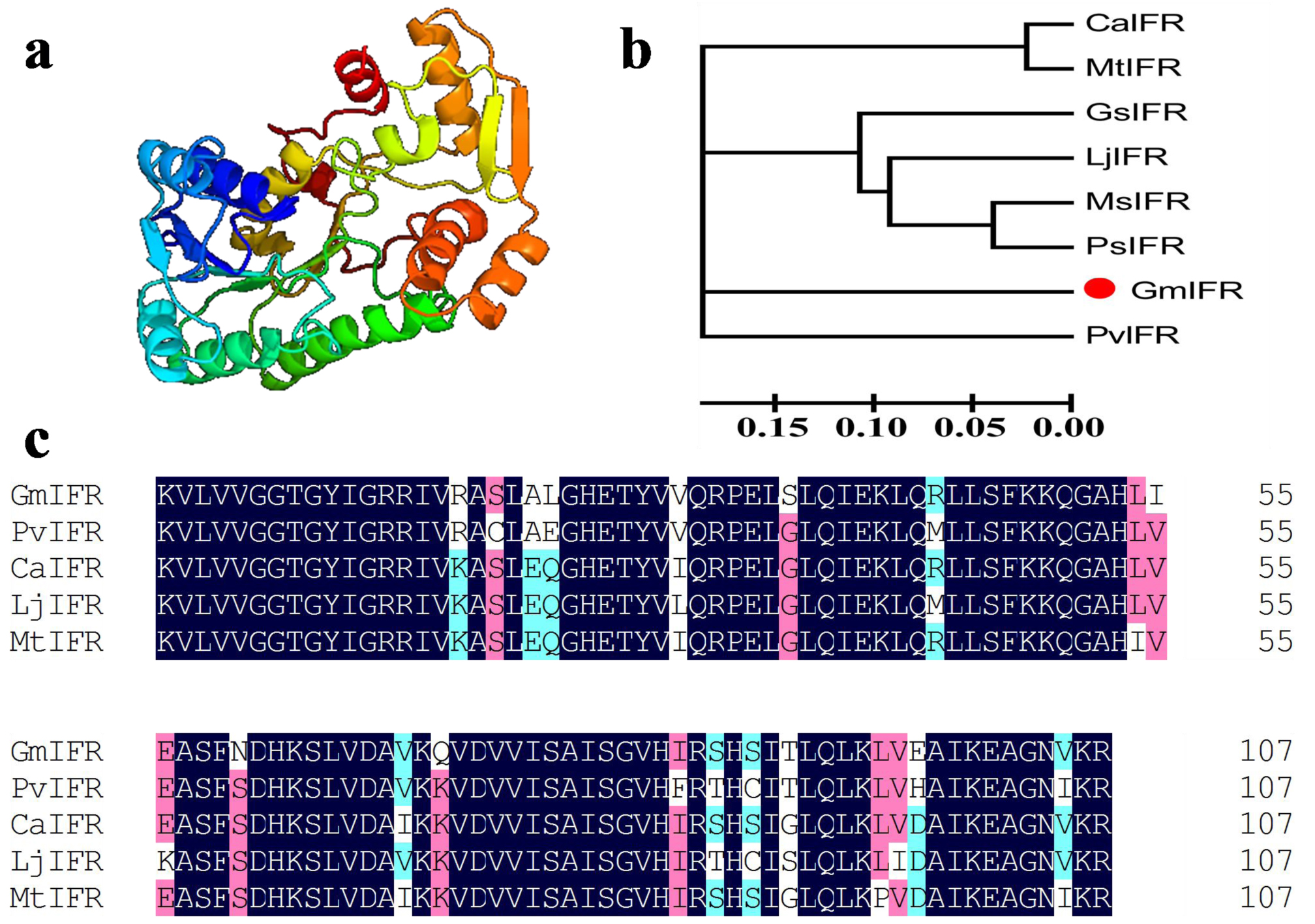

Supplement: Supplementary Figure S2 — Phylogenetic analysis and sequence alignment of GmIFR in the leguminous plants genome. (A) The predicted three-dimensional model of the IFR. (B) Phylogenetic relationships of GmIFR with IFRs proteins; the phylogenetic tree was constructed using amino acid sequences of the IFRs protein from other species. The plant species and GenBank accession numbers were as follows: Glycine max GmIFR (NM_001254100); Phaseolus vulgaris PvIFR (XP_007156276.1), Cicer arietinum CaIFR (XP_004509553.1), Medicago truncatula MtIRF (AFK37791.1), Lotus japonicus LjIFR (BAF34845.1), Glycine soja GsIFR (KHN36092.1), Medicago sativa MsIFR (CAA41106), Pisum sativum PsIFR (P52576.1). (C) The conserved NAD (P) domain of the IFR group proteins. [file Image2.JPEG]
